# Hypoxic-Ischemic Encephalopathy and Beyond: A Pictorial Review of Neuroimaging Findings in Neonatal Encephalopathy

**DOI:** 10.7759/cureus.85625

**Published:** 2025-06-09

**Authors:** Gleidson Silva, Brandon Simons, Kanika Gupta, Jennifer Kucera, Thomas R VerHage, Manish Bajaj, Tushar Chandra

**Affiliations:** 1 Pediatric Radiology, Nemours Children's Hospital, Orlando, USA; 2 Pediatric Radiology, University of Central Florida College of Medicine, Orlando, USA; 3 Radiology, University of Arizona College of Medicine-Tucson, Tucson, USA

**Keywords:** central nervous system infections, hypoxic-ischemic encephalopathy (hie), inborn errors of metabolism, metabolic encephalopathy, myelination patterns, neonatal encephalopathy (ne), neonatal stroke, neuroimaging

## Abstract

Neonatal encephalopathy (NE) encompasses a broad spectrum of neurological dysfunction in newborns, presenting with varying degrees of severity and diverse etiologies. This pictorial review aims to provide an overview of imaging findings associated with NE, highlighting both common and uncommon presentations. By focusing on hypoxic-ischemic encephalopathy (HIE), neonatal stroke, metabolic encephalopathy due to inborn errors of metabolism, central nervous system infections, and structural/genetic causes, this review underscores the importance of accurate diagnosis and management through neuroimaging. Evaluating neonatal imaging for signs of encephalopathy requires meticulous attention to specific characteristics associated with various etiologies. The neonatal brain exhibits distinct myelination patterns, and recognizing normal imaging in full-term and preterm neonates is essential for identifying abnormalities. HIE can manifest with distinct signal changes depending on the injury pattern, which can be either severe total hypoxia, prolonged partial hypoxia, or a combination of these. Magnetic resonance imaging (MRI), particularly diffusion-weighted imaging (DWI), is helpful in detecting these changes. Metabolic encephalopathies often present with overlapping imaging features, and identifying the primary pattern of involvement, such as white matter, deep gray matter, or a combination, serves as a starting point for differentiating etiologies. In cases of neonatal brain infections, contrast-enhanced MRI can detect early involvement of the meninges, ependymal lining, and brain parenchyma, with DWI being particularly useful for identifying ischemic areas and purulent accumulations that may not be visible with other imaging modalities. Structural and genetic causes of NE can lead to specific imaging findings that are crucial for accurate diagnosis and tailored management.

## Introduction and background

**Figure 1:**
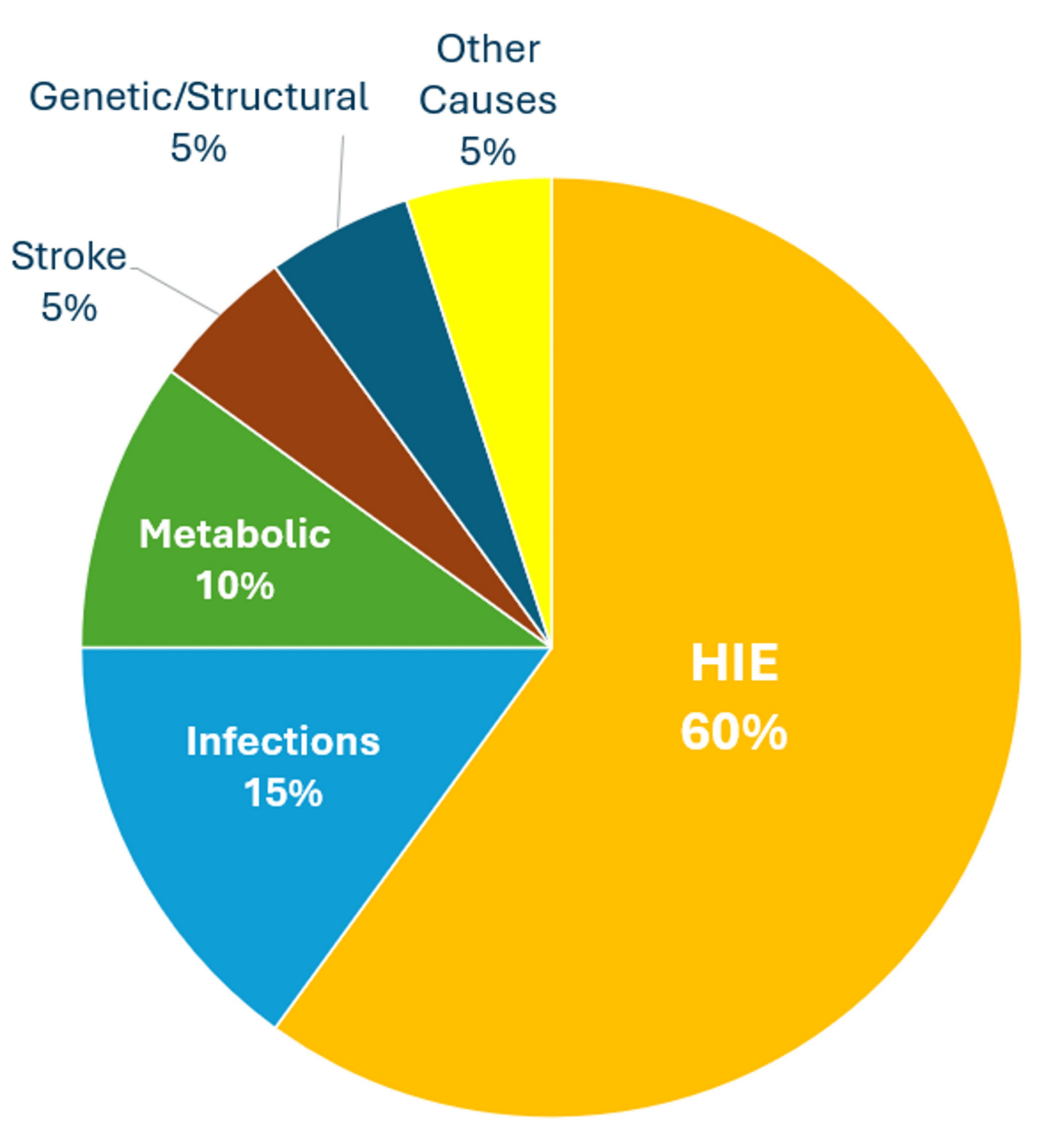
Pie chart illustrating the estimated causes of neonatal encephalopathy Pie chart created by the authors based on data from [2]. HIE: hypoxic-ischemic encephalopathy

Neonatal encephalopathy (NE) is a clinical syndrome affecting newborns, characterized by disturbed neurological function. This condition can manifest through mild irritability and feeding issues to severe cases involving seizures and coma in infants born at or beyond 35 weeks of gestation [1]. The causes of NE are diverse, including hypoxic-ischemic events, infections, metabolic disorders, stroke, and genetic abnormalities (Figure 1). Early and accurate diagnosis and management are essential for effective treatment, prognosis, and genetic counseling [2]. Magnetic resonance imaging (MRI) provides high-resolution structural, diffusion-weighted, and spectroscopic evaluation of the neonatal brain. This review categorizes and illustrates the MRI findings of hypoxic-ischemic encephalopathy (HIE) and non-HIE causes of NE, including multiple examples for each.

## Review

Methods

A retrospective review of an institutional picture archiving and communication system (PACS) was conducted to identify term neonates (≤30 days old) who presented with clinical signs of NE. Inclusion criteria encompassed cases with documented HIE, seizures, feeding difficulties, altered mental status, or respiratory distress. A targeted keyword-based search strategy was used to ensure comprehensive and consistent case identification. Relevant imaging and clinical data were reviewed by the authors. All figures and illustrations were originally created by the authors to enhance visual clarity and support the educational objectives of this article.

Patterns of brain injury and normal appearance of a neonatal brain on MRI

**Figure 2:**
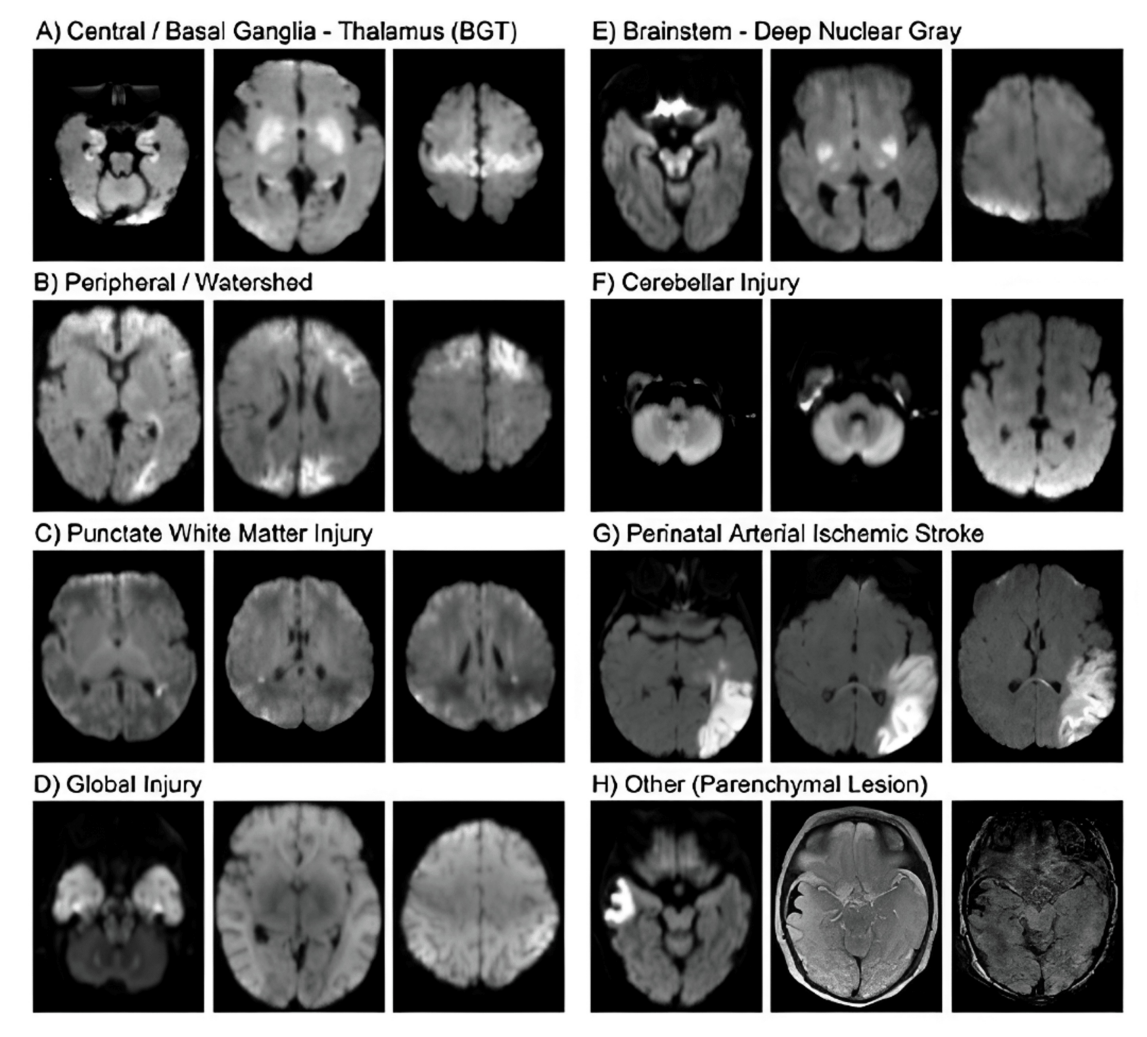
Patterns of brain injury in neonates on MRI DWI sequences (a) Central/basal ganglia-thalamus (BGT); (b) Peripheral/watershed; (c) Punctate white matter injury; (d) Global injury; (e) Brainstem-deep nuclear gray; (f) Cerebellar injury; (g) Perinatal ischemic stroke; (h) Other (parenchymal lesion)—subpial hemorrhage. DWI: diffusion-weighted imaging Reproduced from Seminars in Fetal and Neonatal Medicine, Volume 26, Issue 5, Wisnowski, JL et al., Neuroimaging in the term newborn with neonatal encephalopathy, 101304, Copyright (2021), with permission from Elsevier [3].

**Figure 3:**
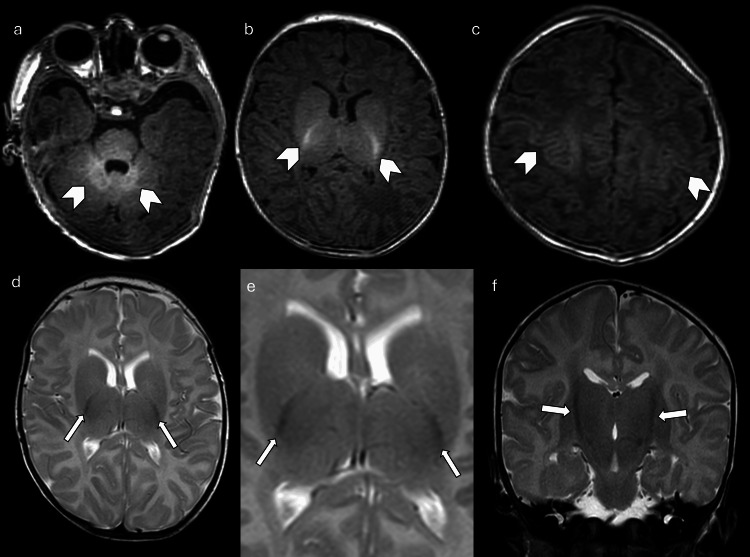
Normal myelination at birth (a), (b), and (c): Axial T1W images showing T1 shortening in the dorsal brainstem, posterior limb of internal capsule, lateral thalamus, central centrum semiovale, and perirolandic region (arrowheads) in term neonates serve as essential references to identify pathological deviations. (d), (e), and (f): Axial and coronal T2 images demonstrate T2 hypointensity in the posterior limb of the internal capsule (arrows) that corresponds to areas of normal early myelination. Images obtained by the authors from the institutional PACS. All images are de-identified and used in accordance with institutional policy. T1W: T1-weighted; PACS: picture archiving and communication system

Recognizing the most common patterns of brain injury (Figure 2) [3] and the normal appearance of a neonatal brain on MRI studies (Figure 3) [4,5] is crucial for identifying the injury and narrowing down the possible etiologies. Normal myelination in neonates starts in the brainstem and cerebellum around mid-gestation and progresses caudally to rostrally. By term, myelination is seen in the posterior limb of the internal capsule, optic radiations, and cerebellar white matter. Postnatally, it continues in the cerebral hemispheres, especially the frontal and occipital lobes, during the first year [4]. Early identification of the specific etiology allows for timely medical intervention, which can significantly improve outcomes. Different etiologies may require different treatment approaches, and accurate recognition helps healthcare providers develop personalized treatment plans. For instance, identifying a pattern indicative of HIE can direct clinicians to investigate perinatal asphyxia as the primary cause, while recognizing patterns associated with metabolic encephalopathies can prompt further evaluation for inborn errors of metabolism. This targeted approach not only aids in diagnosis but also enhances the precision of subsequent management strategies. Additionally, identifying common patterns aids in stratifying patients for clinical trials, ensuring that studies are appropriately targeted to specific types of injuries, leading to more effective treatments [5]. 

HIE

**Figure 4:**
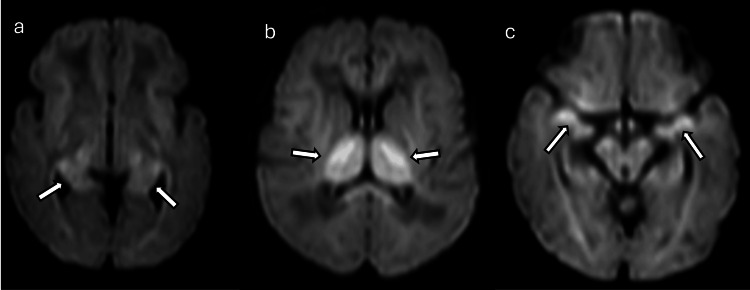
A four-day-old patient with hypoxic ischemic encephalopathy (a), (b), and (c): Axial DWI sequences demonstrate central/basal ganglia–thalamus pattern injury with symmetric restricted diffusion involving both thalami, splenium, posterior limb of internal capsule, corticospinal tract, and hippocampi (arrows). Images obtained by the authors from the institutional PACS. All images are de-identified and used in accordance with institutional policy. DWI: diffusion-weighted imaging; PACS: picture archiving and communication system

**Figure 5:**
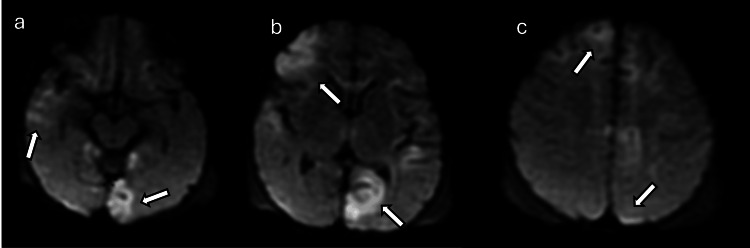
A five-day-old patient with seizures (a), (b), and (c): Axial DWI images show multifocal areas of restricted diffusion throughout the cerebral cortex (arrows) with sparing of the deep gray matter (watershed pattern). Images obtained by the authors from the institutional PACS. All images are de-identified and used in accordance with institutional policy. DWI: diffusion-weighted imaging; PACS: picture archiving and communication system

**Figure 6:**
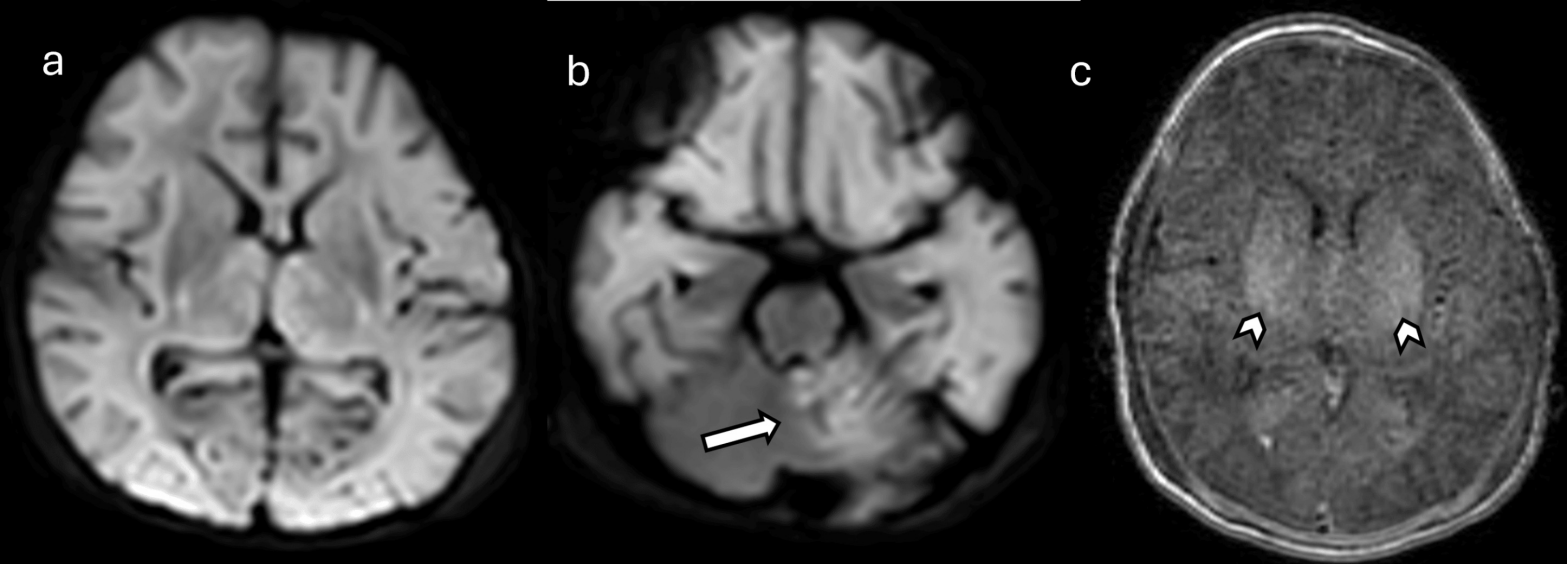
A four-day-old patient with a history of hypoxic ischemic encephalopathy, status post rewarming (a) and (b) axial DWI and (c) axial T1W images showing global injury pattern with widespread, extensive restricted diffusion involving the gray and white matter of both cerebral hemispheres, corpus callosum thalami, the corticospinal tracts, basal ganglia, and the left cerebellar hemisphere (arrow). Note the internal capsule posterior limb signal loss on T1W (arrowheads). Images obtained by the authors from the institutional PACS. All images are de-identified and used in accordance with institutional policy. DWI: diffusion-weighted imaging; T1W: T1-weighted; PACS: picture archiving and communication system

HIE is one of the most common causes of NE, accounting for approximately 60% of cases [2]. HIE results from a reduction in blood and oxygen supply to the brain around the time of birth. HIE can present with varying degrees of severity, and its diagnosis often relies on MRI, especially DWI. These imaging modalities help identify distinct patterns of brain injury, which are crucial for determining the extent of damage and guiding therapeutic interventions [6-9]. Acute profound injury may involve bilateral regions of the posterior limb of the internal capsule, basal ganglia, thalami, and perirolandic cortex (Figure 4). Partial prolonged injury typically shows a watershed pattern between the anterior and middle cerebral arteries, decreased N-acetylaspartate (NAA), and elevated lactate levels (Figure 5). Severe HIE can present with selective deep gray matter damage, global cortical restricted diffusion, and/or cerebral edema, potentially linked to acute sentinel events like placental abruption (Figure 6) [3, 10, 11].

Central nervous system infections

**Figure 7:**
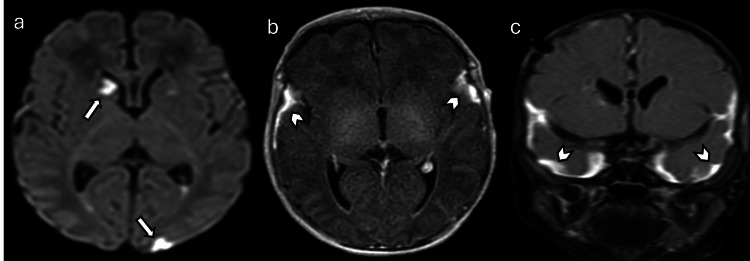
GBS meningitis in an 11-day-old baby with history of GBS infection (a) Axial DWI, (b) axial T1W post-contrast, and (c) FLAIR postcontrast MRI showed multifocal areas of restricted diffusion in the brain parenchyma (arrows). Linear dural enhancement overlying the inferior temporal lobes, the operculum, and the occipital lobes (arrowheads), consistent with meningeal inflammation and/or subdural empyema. Images obtained by the authors from the institutional PACS at Nemours Children's Hospital. All images are de-identified and used in accordance with institutional policy. GBS: Group B streptococcal; DWI: diffusion-weighted imaging; T1W: T1-weighted; FLAIR: fluid-attenuated inversion recovery; PACS: picture archiving and communication system

**Figure 8:**
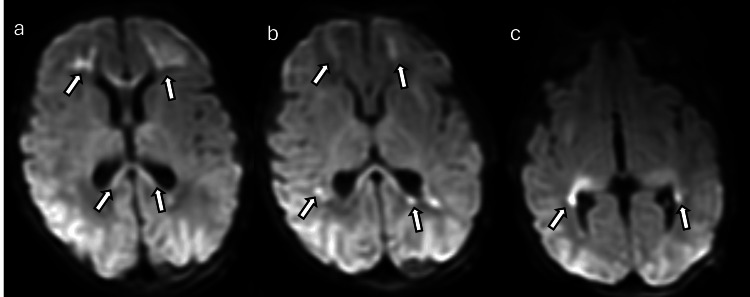
Human parechovirus meningoencephalitis in a 12-day-old female patient with sepsis and seizures (a), (b), and (c): Axial DWI images demonstrate multifocal frontal-predominant subcortical white matter and callosal involvement with associated low diffusivity (arrows) and thalamic involvement. Images obtained by the authors from the institutional PACS. All images are de-identified and used in accordance with institutional policy. DWI: diffusion-weighted imaging; PACS: picture archiving and communication system

Central nervous system infections contribute to about 15% of NE cases. Pathogens can cause inflammation and damage to brain tissue, leading to NE. A common early finding is enhancement of the meninges, ependymal lining, and brain parenchyma (Figure 7). DWI is particularly useful in identifying ischemic areas and purulent accumulations that may not be visible with other imaging modalities (Figure 8) [12,13].

Metabolic encephalopathies

**Figure 9:**
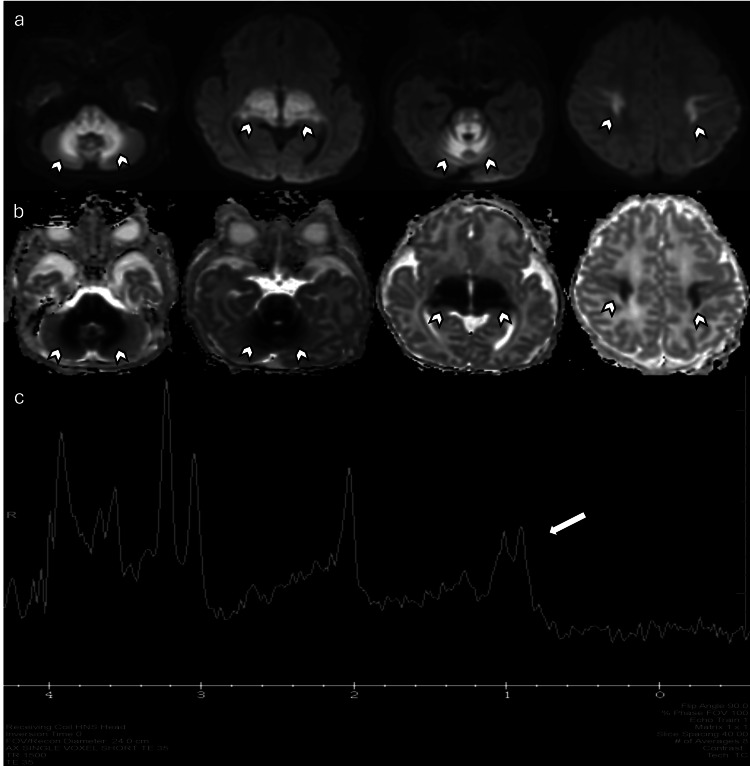
Maple syrup urine disease in a seven-day-old female patient with poor feeding and increasing lethargy (a) Axial DWI and (b) axial ADC maps demonstrate restricted diffusion of the spinal cord, brainstem, cerebellar white matter, thalamus, and corticospinal tract (arrowheads). (c) MRS at TE 35 ms shows an abnormal peak seen at 0.9 PPM, mostly consistent with a high branched-chain amino acid peak. Images obtained by the authors from the institutional PACS. All images are de-identified and used in accordance with institutional policy. DWI: diffusion-weighted imaging; ADC: apparent diffusion coefficient; MRS: magnetic resonance spectroscopy; TE: echo time; PPM: parts per million; PACS: picture archiving and communication system

**Figure 10:**
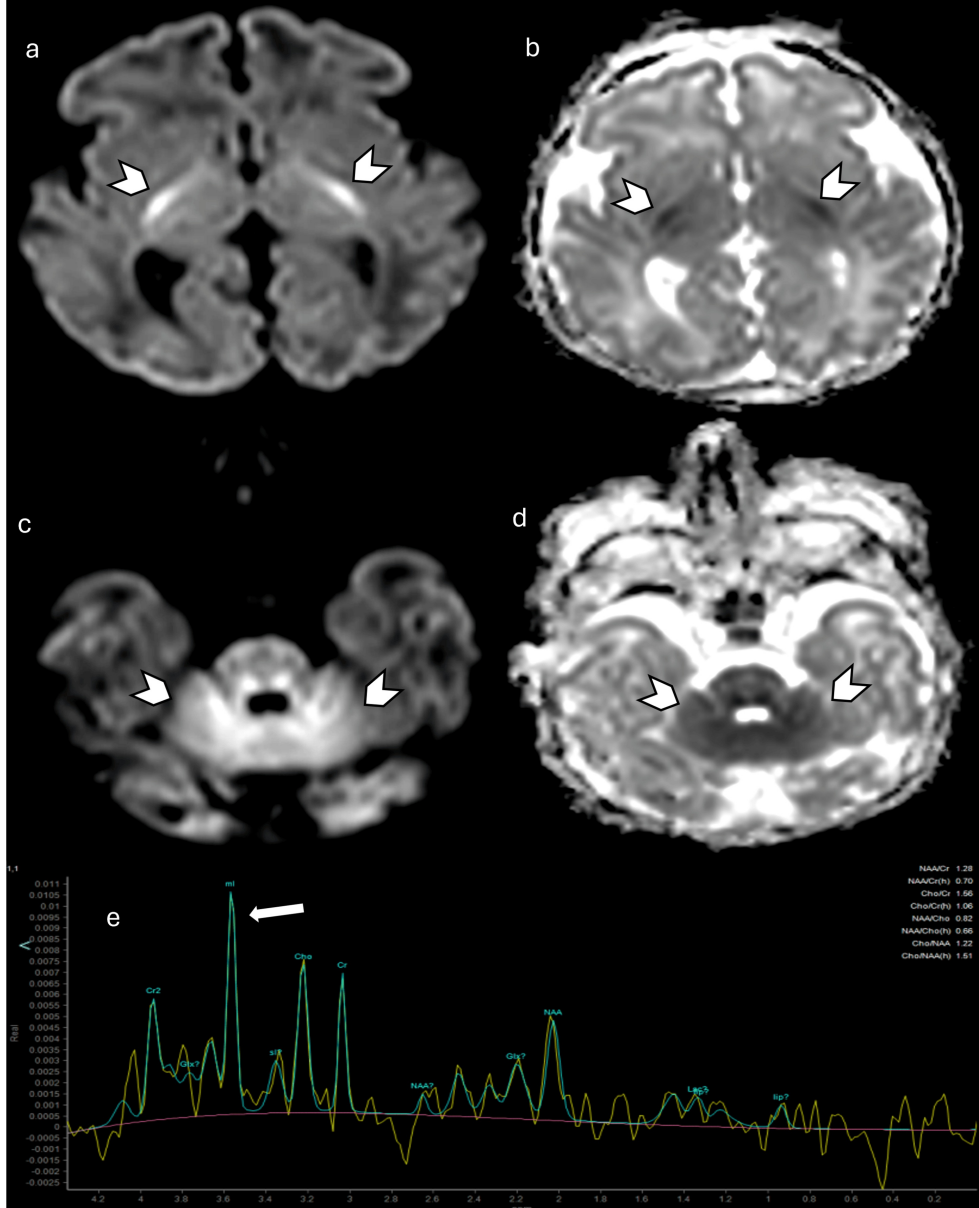
Nonketotic hyperglycemia in a three-day-old patient presenting with hypotonia; EEG showing burst suppression, and hiccups. (a) and (c) Axial DWI, (b) and (d) axial ADC map showing restricted diffusion in the corticospinal tracts, dorsal brainstem, cerebellar peduncles, and cerebellar white matter (arrowheads). (e) MRS showing abnormal peak in the white matter and basal ganglia at 3.56 ppm at TE 144 ms, likely representing elevated glycine peak (arrow). Images obtained by the authors from the institutional PACS. All images are de-identified and used in accordance with institutional policy. DWI: diffusion-weighted imaging; ADC: apparent diffusion coefficient; MRS: magnetic resonance spectroscopy; TE: echo time; PPM: parts per million; PACS: picture archiving and communication system

Metabolic encephalopathies account for approximately 10% of NE cases and often result from inborn errors of metabolism. These conditions can lead to the accumulation of toxic substances in the brain, causing widespread neurological damage. Certain metabolic conditions exhibit distinctive signal abnormalities affecting the white and/or gray matter, which helps in identifying the specific disorder, such as maple syrup urine disease (Figure 9) and nonketotic hyperglycemia (Figure 10) [14,15].

Neonatal stroke

**Figure 11:**
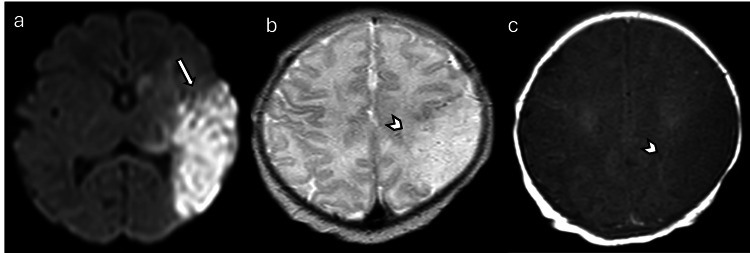
Perinatal stroke in a two-day-old patient with seizures (a) Axial DWI shows an area of restricted diffusion involving the left MCA distribution, including the left temporal lobe and watershed zones in the left parietal region and the posterior aspect of the left frontal lobe (arrow). (b) Axial T2W image demonstrates edema (arrowhead), and (c) axial T1W image shows loss of the normal T1 hyperintensity in the left peri-Rolandic area (arrowhead). Images obtained by the authors from the institutional PACS. All images are de-identified and used in accordance with institutional policy. DWI: diffusion-weighted imaging; MCA: middle cerebral artery; T2W: T2-weighted; T1W: T1-weighted; PACS: picture archiving and communication system

Neonatal stroke represents about 5% of NE cases. Perinatal arterial ischemic strokes can lead to acute ischemic lesions localized within a cerebral artery distribution, most commonly the middle cerebral artery (MCA) [2, 3, 8, 10, 11]. The most common MRI findings are unilateral diffusion restriction in the MCA or anterior cerebral artery (ACA) territory and usually spare deep gray matter, in contrast to HIE. Additionally, T2/fluid-attenuated inversion recovery (FLAIR) signal changes can provide valuable information regarding the timing of the injury, distinguishing between acute and subacute phases (Figure 11) [16-18].

Genetic and structural disorders

**Figure 12:**
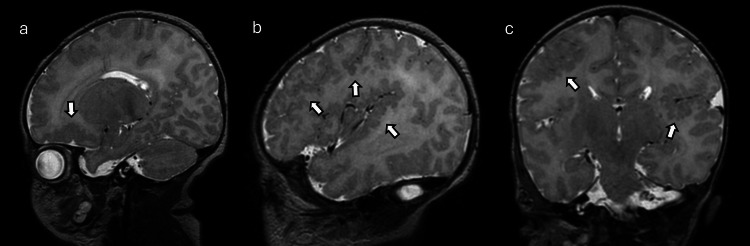
Cabezas X-linked intellectual disability in a nine-day-old boy with feeding difficulty (a), (b) sagittal, and (c) coronal T2W MRI showing extensive polymicrogyria involving bilateral perisylvian cortex and bilateral frontal lobes (arrows). DWI and ADC maps were normal. Images obtained by the authors from the institutional PACS. All images are de-identified and used in accordance with institutional policy. T2W: T2-weighted; DWI: diffusion-weighted imaging; ADC: apparent diffusion coefficient; PACS: picture archiving and communication system

Genetic and structural disorders contribute to approximately 5% of NE cases. These include congenital brain malformations such as heterotopias, schizencephaly, and lissencephaly, as well as genetic syndromes that affect brain development and function. Imaging can reveal structural abnormalities indicative of these conditions, aiding in early diagnosis and management (Figure 12) [19].

Other causes

**Figure 13:**
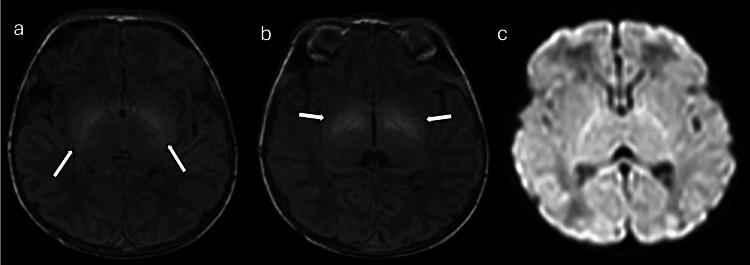
Kernicterus in a seven-day-old with hyperbilirubinemia (a) and (b) T1W MRI, showing a mild increase in T1 signal involving the bilateral globus pallidus and subthalamic nuclei (arrows), and (c) DWI with no evidence of restricted diffusion. Images obtained by the authors from the institutional PACS. All images are de-identified and used in accordance with institutional policy. T1W: T1-weighted; DWI: diffusion-weighted imaging; PACS: picture archiving and communication system

**Figure 14:**
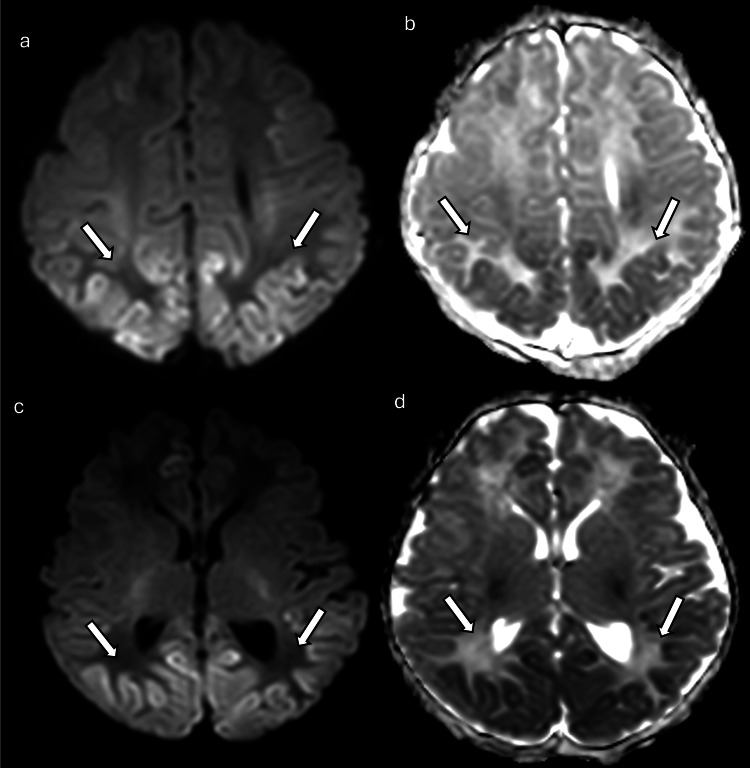
Hypoglycemic encephalopathy in a one-day-old newborn with seizures, lethargy, decreased intake, and low blood sugar (a) and (c) DWI and (b) and (d) ADC maps show areas of restricted diffusion involving bilateral occipital lobes, bilateral posterior temporal lobes, the superior aspect of the vermis of the cerebellum, the dorsal lateral aspect of the midbrain bilaterally, bilateral thalami, bilateral hippocampi, the right side of the cingulate gyrus, and the cortex/subcortical region of bilateral frontotemporal regions (arrows). Images obtained by the authors from the institutional PACS. All images are de-identified and used in accordance with institutional policy. DWI: diffusion-weighted imaging; ADC: apparent diffusion coefficient; PACS: picture archiving and communication system

Other causes make up the remaining 5% of NE cases. These can include maternal drug use, severe jaundice leading to bilirubin encephalopathy (Figure 13), and hypoglycemia (Figure 14). Each of these causes presents unique imaging features that are essential for accurate diagnosis and management [20,21].

## Conclusions

Accurate diagnosis of NE is essential for effective management and treatment. Differentiating normal imaging patterns from pathological findings is crucial, especially given the specific myelination patterns seen in newborns, which can be mistaken for hypoxic injury. HIE presents distinct imaging features based on the type of injury, such as changes in the basal ganglia, thalamus, or cortical areas. Infections can lead to diffuse or focal abnormalities, often involving the meninges or parenchyma. Metabolic encephalopathies, while having overlapping imaging features, can be categorized primarily by white matter or gray matter involvement. Neonatal stroke typically affects arterial territories, with the middle cerebral artery being most involved. Genetic and structural abnormalities may manifest as malformations or dysgenesis, impacting various brain regions. Other causes, such as toxic exposures, can result in varied imaging findings. It is important for radiologists to be aware of these different entities to make a timely and accurate diagnosis to guide clinical management.
